# Predicting prolonged postoperative length of stay risk in patients undergoing lumbar fusion surgery: Development and assessment of a novel predictive nomogram

**DOI:** 10.3389/fsurg.2022.925354

**Published:** 2022-08-16

**Authors:** Chen-Xin Lu, Zhi-Bin Huang, Xiao-Mei Chen, Xiao-Dan Wu

**Affiliations:** ^1^ Department of Anesthesiology, Fuzhou Second Hospital, Fuzhou, China; ^2^ Department of Anesthesiology, Fujian Provincial Hospital, Shengli Clinical Medical College of Fujian Medical University, Fujian Medical University, Fuzhou, China

**Keywords:** PLOS, lumbar fusion surgery, nomogram, predictive model, LASSO regression

## Abstract

**Objective:**

The purpose of this study was to develop and internally validate a prediction nomogram model in patients undergoing lumbar fusion surgery.

**Methods:**

A total of 310 patients undergoing lumbar fusion surgery were reviewed, and the median and quartile interval were used to describe postoperative length of stay (PLOS). Patients with PLOS > P_75_ were defined as prolonged PLOS. The least absolute shrinkage and selection operator (LASSO) regression was used to filter variables for building the prolonged PLOS risk model. Multivariable logistic regression analysis was applied to build a predictive model using the variables selected in the LASSO regression model. The area under the ROC curve (AUC) of the predicting model was calculated and significant test was performed. The Kappa consistency test between the predictive model and the actual diagnosis was performed. Discrimination, calibration, and the clinical usefulness of the predicting model were assessed using the C-index, calibration plot, and decision curve analysis. Internal validation was assessed using the bootstrapping validation.

**Results:**

According to the interquartile range of PLOS in a total of 310 patients, the PLOS of 235 patients was ≤P_75_ (7 days) (normal PLOS), and the PLOS of 75 patients was > P_75_ (prolonged PLOS). The LASSO selected predictors that were used to build the prediction nomogram included BMI, diabetes, hypertension, duration of surgery, duration of anesthesia, anesthesia type, intraoperative blood loss, sufentanil for postoperative analgesia, and postoperative complication. The model displayed good discrimination with an AUC value of 0.807 (95% CI: 0.758–0.849, *P *< 0.001), a Kappa value of 0.5186 (cutoff value, 0.2445, *P* < 0.001), and good calibration. A high C-index value of 0.776 could still be reached in the interval validation. Decision curve analysis showed that the prolonged PLOS nomogram was clinically useful when intervention was decided at the prolonged PLOS possibility threshold of 3%.

**Conclusions:**

This study developed a novel nomogram with a relatively good accuracy to help clinicians access the risk of prolonged PLOS in lumbar fusion surgery patients. By an estimate of individual risk, surgeons and anesthesiologists may shorten PLOS and accelerate postoperative recovery of lumbar fusion surgery through more accurate individualized treatment.

## Introduction

In recent years, with the prevalence of the concept of enhanced recovery after surgery (ERAS), clinicians related to the perioperative period are gradually beginning to pay attention to the implementation of this concept. The essence of ERAS is to improve the preoperative state of patients, ensure the safety of patients, minimize perioperative stress response, shorten the postoperative length of stay (PLOS), and accelerate the recovery of patients (1, 2).

In spine surgery, lumbar fusion surgery is one of the common surgical procedures. Studies have shown that the PLOS of lumbar fusion ranges between 3 and 6.7 days (3). The prolongation of PLOS not only does not meet the requirements of ERAS but also is disadvantageous to the patients, causing physical, mental, and financial burden for them. Prolonged PLOS is associated with many perioperative adverse outcomes, such as increasing the risk of hospital-acquired infection and deep venous thrombosis, and even endangering the lives of patients (4, 5). During the perioperative period, the PLOS of patients is affected by many factors (6–11). As a visual presentation of the relationship between risk factors and outcome, predictive nomogram is favored by clinicians. However, there is no nomogram for predicting the risk of prolonged PLOS in lumbar fusion surgery.

The aim of this study was to develop a valid but simple prediction nomogram model in lumbar fusion surgery to assess the risk of prolonged PLOS using only those clinical variables easily available.

## Patients and methods

### Patients

Research approval was obtained from the Ethics Committee of Fuzhou Second Hospital. The subjects were 310 patients who underwent lumbar fusion surgery in the Fuzhou second Hospital from 1 January 2019 to 1 December 2019. The median and quartile interval were used to describe PLOS. Patients with PLOS > P_75_ were defined as prolonged PLOS (12, 13). According to whether PLOS was prolonged, the patients were divided into a case group and a control group. A total of 75 patients with prolonged PLOS were included in the case group, and 235 patients were included in the control group. Data such as demographic, preoperative data (ASA class, diabetes, hypertension, number of comorbidities), intraoperative data (duration of surgery, anesthesia type, fluid infusion volume, blood transfusion, and blood loss volume), and postoperative data (PLOS, analgesia dosage of sufentanil, and postoperative complications) were collected from medical records.

### Inclusion and exclusion criteria

We included patients aged 18 years and older undergoing the elective lumbar fusion surgery. Exclusion criteria: (1) patients treated with minimally invasive technique or requiring more than three segmental internal fixation; (2) trauma patients; and (3) patients with spinal tumors, abscesses, spinal deformities (i.e. scoliosis and kyphosis), spinal fractures, vertebroplasty, osteomyelitis, and cauda equina syndrome.

### Statistical analysis

All data were expressed as count (%). Statistical analysis was performed using the R software (Version 4.1.3; https://www.R-project.org), IBM SPSS version 23.0, and MedCalc (Version 19.2; https://www.medcalc.org).

The least absolute shrinkage and selection operator (LASSO) method was used to select the optimal predictive variables in risk factors from the patients undergoing lumbar fusion surgery (14, 15). Variables with nonzero coefficients in the LASSO regression model were selected (16). Then, multivariable logistic regression analysis was used to build a predicting model by incorporating the variables selected in the LASSO regression model (17). The variables were considered as odds ratio (OR) having 95% confidence interval (CI) and as *P*-value. The statistical significance levels were all two-sided. The area under the ROC curve (AUC) of the predicting model was calculated and significant test was performed by using MedCalc software. The Kappa consistency test between the predictive model and the actual diagnosis was performed by using IBM SPSS version 23.0. All potential predictors selected in the LASSO regression model were applied to develop a predicting model nomogram for prolonged PLOS risk.

Calibration curves were plotted to assess the calibration of the prolonged PLOS risk nomogram. The prolonged PLOS risk nomogram was subjected to bootstrapping validation (1,000 bootstrap resamples) to calculate a relatively corrected C-index (18). Decision curve analysis was conducted to determine the clinical usefulness of the prolonged PLOS risk nomogram by quantifying the net benefits at different threshold probabilities in the lumbar fusion surgery cohort. The net benefit was calculated by subtracting the proportion of all patients who were false positive from the proportion of those patients who were true positive and by weighing the relative harm of forgoing interventions compared with the negative consequences of an unnecessary intervention (19, 20).

## Results

### Definition of prolonged PLOS

According to the inclusion and exclusion criteria, 310 patients undergoing lumbar fusion surgery were analyzed in this study. PLOS was treated as an outcome variable and tested for normality. We found that the PLOS data did not conform to the normal distribution (*P* < 0.001). Therefore, the median and quartile intervals were used to describe the PLOS. The median PLOS was 6 days and P_75_ was 7 days. The patients with PLOS > P_75_ were defined as prolonged PLOS (12, 13).

### Patients' characteristics

**Figure 1:**
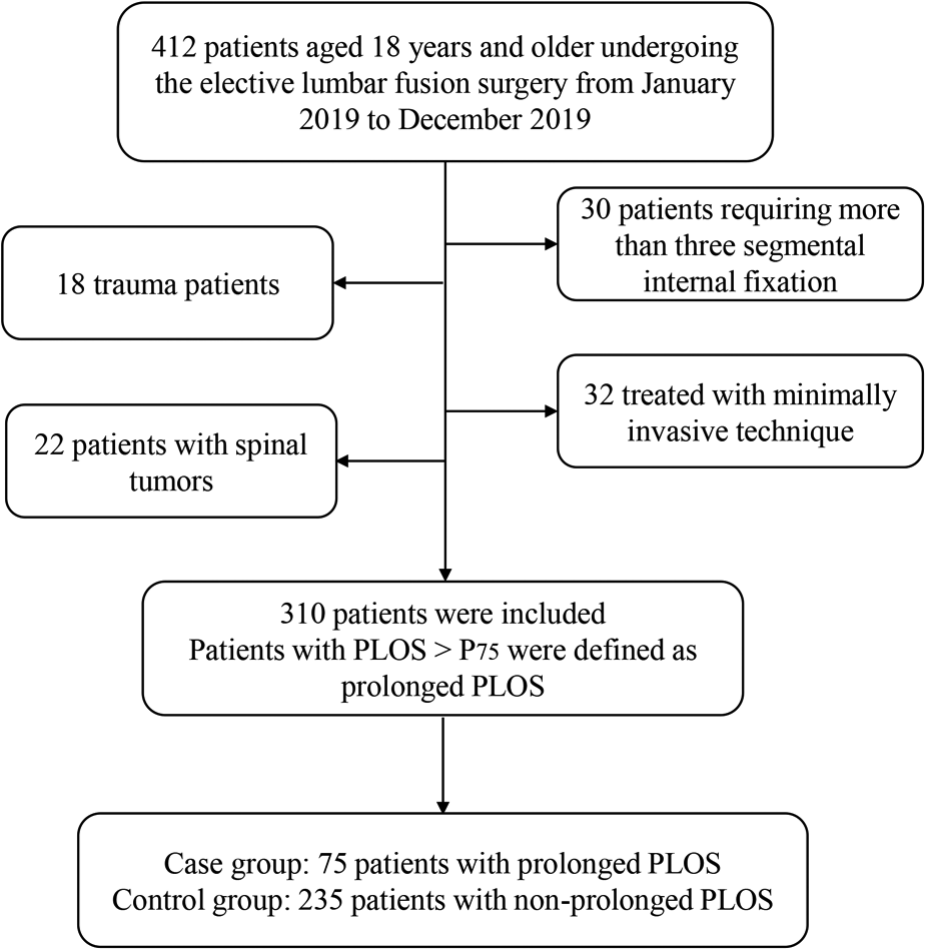
Study flowchart. PLOS, postoperative length of stay.

According to the interquartile range of PLOS in a total of 310 patients, the PLOS of 235 patients was ≤P75 (7 days) (normal PLOS), and the PLOS of 75 patients was >P75 (prolonged PLOS) (Figure 1). All data of patients, including demographic and perioperative clinical features in the two groups, are given in Table 1.

**Table 1:** Differences between demographic and clinical characteristics of prolonged PLOS and non-prolonged PLOS groups.

Variables	Prolonged PLOS		Standardize diff.	*P*-value
	No (*n* = 235)	Yes (*n* = 75)		
Age (years)			0.1 (−0.2, 0.3)	0.508
<65	154 (65.5%)	46 (61.3%)		
≥65	81 (34.5%)	29 (38.7%)		
BMI (kg/m^2^)			0.2 (−0.0, 0.5)	0.070
<28	217 (92.3%)	64 (85.3%)		
≥28	18 (7.7%)	11 (14.7%)		
ASA class			0.0 (−0.2, 0.3)	0.819
1–2	224 (95.3%)	71 (94.7%)		
3–4	11 (4.7%)	4 (5.3%)		
Comorbidities			0.2 (−0.1, 0.4)	0.169
<3	170 (72.3%)	48 (64.0%)		
≥3	65 (27.7%)	27 (36.0%)		
Diabetes			0.2 (−0.1, 0.5)	0.108
No	203 (86.4%)	59 (78.7%)		
Yes	32 (13.6%)	16 (21.3%)		
Hypertension			0.0 (−0.2, 0.3)	0.865
No	151 (64.3%)	49 (65.3%)		
Yes	84 (35.7%)	26 (34.7%)		
Duration of surgery (hours)			0.6 (0.3, 0.8)	<0.001
<2	86 (36.6%)	10 (13.3%)		
≥2	149 (63.4%)	65 (86.7%)		
Duration of anesthesia (h)			0.4 (0.2, 0.7)	0.004
<2	46 (19.6%)	4 (5.3%)		
≥2	189 (80.4%)	71 (94.7%)		
Anesthesia type			0.4 (0.1, 0.6)	0.004
CIIA	170 (72.3%)	41 (54.7%)		
TIA	65 (27.7%)	34 (45.3%)		
Intraoperative blood loss (ml)			0.3 (0.1, 0.6)	0.010
<500	218 (92.8%)	62 (82.7%)		
≥500	17 (7.2%)	13 (17.3%)		
Blood transfusion			0.1 (−0.1, 0.4)	0.276
No	219 (93.2%)	67 (89.3%)		
Yes	16 (6.8%)	8 (10.7%)		
Intraoperative fluid infusion volume (ml/kg)			0.3 (0.1, 0.6)	0.148
<17.2	61 (26.0%)	11 (14.7%)		
≥17.2, <22.2	62 (26.4%)	18 (24.0%)		
≥22.2, <28.6	57 (24.3%)	24 (32.0%)		
≥28.6	55 (23.4%)	22 (29.3%)		
Intraoperative urine volume (ml/kg/h)			0.2 (−0.1, 0.5)	0.532
<0.71	59 (25.1%)	20 (26.7%)		
≥0.71, <1.27	56 (23.8%)	19 (25.3%)		
≥1.27, <2.32	57 (24.3%)	22 (29.3%)		
≥2.32	63 (26.8%)	14 (18.7%)		
Sufentanil for postoperative analgesia (μg/kg)			0.2 (−0.0, 0.5)	0.054
<2	206 (87.7%)	59 (78.7%)		
≥2	29 (12.3%)	16 (21.3%)		
Postoperative complication			1.0 (0.7, 1.2)	<0.001
No	232 (98.7%)	49 (65.3%)		
Yes	3 (1.3%)	26 (34.7%)		

PLOS, postoperative length of stay; BMI, body mass index; ASA, American Society of Anesthesiologists; CIIA, combined intravenous and inhaled anesthesia; TIA, total intravenous anesthesia.

### Variables selection

**Figure 2:**
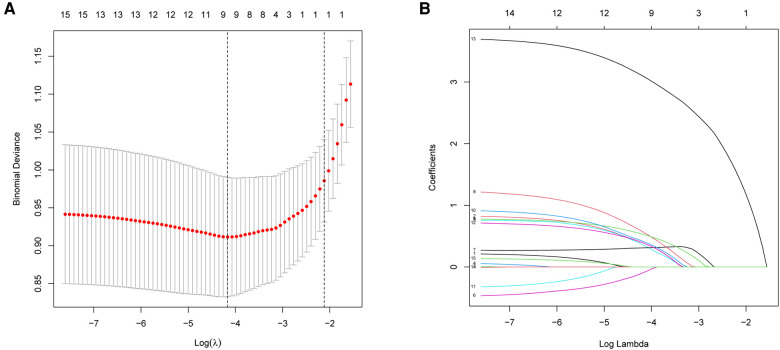
Demographic and clinical feature selection using the LASSO binary logistic regression model. (**A**) Optimal parameter (lambda) selection in the LASSO model used 5-fold cross-validation *via* minimum criteria. The partial likelihood deviance (binomial deviance) curve was plotted versus log (lambda). Dotted vertical lines were drawn at the optimal values by using the minimum criteria and the 1 SE of the minimum criteria (the 1-SE criteria). (**B**) LASSO coefficient profiles of the 15 features. A coefficient profile plot was produced against the log (lambda) sequence. A vertical line was drawn at the value selected using 5-fold cross-validation, where optimal lambda resulted in five features with nonzero coefficients. LASSO, least absolute shrinkage and selection operator; SE, standard error.

Of demographic and perioperative clinical features, 15 variables were reduced to 9 potential predictors on the basis of 310 patients in the cohort with nonzero coefficients in the LASSO regression model (Figures 2A,B). These variables included BMI, diabetes, hypertension, duration of surgery, duration of anesthesia, anesthesia type, intraoperative blood loss, sufentanil for postoperative analgesia, and postoperative complication (Table 2).

**Table 2:** Prediction factors for prolonged PLOS in lumbar fusion surgery.

Intercept and variable	Prediction model		
	*Β*	Odds ratio (95% CI)	*P*-value
BMI	−0.684	0.504 (0.191–1.331)	0.167
Diabetes	−0.778	0.459 (0.198–1.068)	0.071
Hypertension	−0.458	1.581 (0.762–3.282)	0.219
Duration of operation	−0.282	0.754 (0.287–1.986)	0.568
Duration of anesthesia	−1.165	0.312 (0.068–1.437)	0.135
Anesthesia type	−0.772	0.462 (0.243–0.879)	0.019
Estimated blood loss	−0.730	0.482 (0.186–1.249)	0.133
Dose of sufentanil for postoperative analgesia	−0.714	0.490 (0.215–1.118)	0.090
Postoperative complication	−3.659	0.026 (0.007–0.092)	0.000
Intercept	4.973		

CI, confidence interval; PLOS, postoperative length of stay; BMI, body mass index. β is the regression coefficient.

### Development of an individualized prediction model

**Figure 3:**
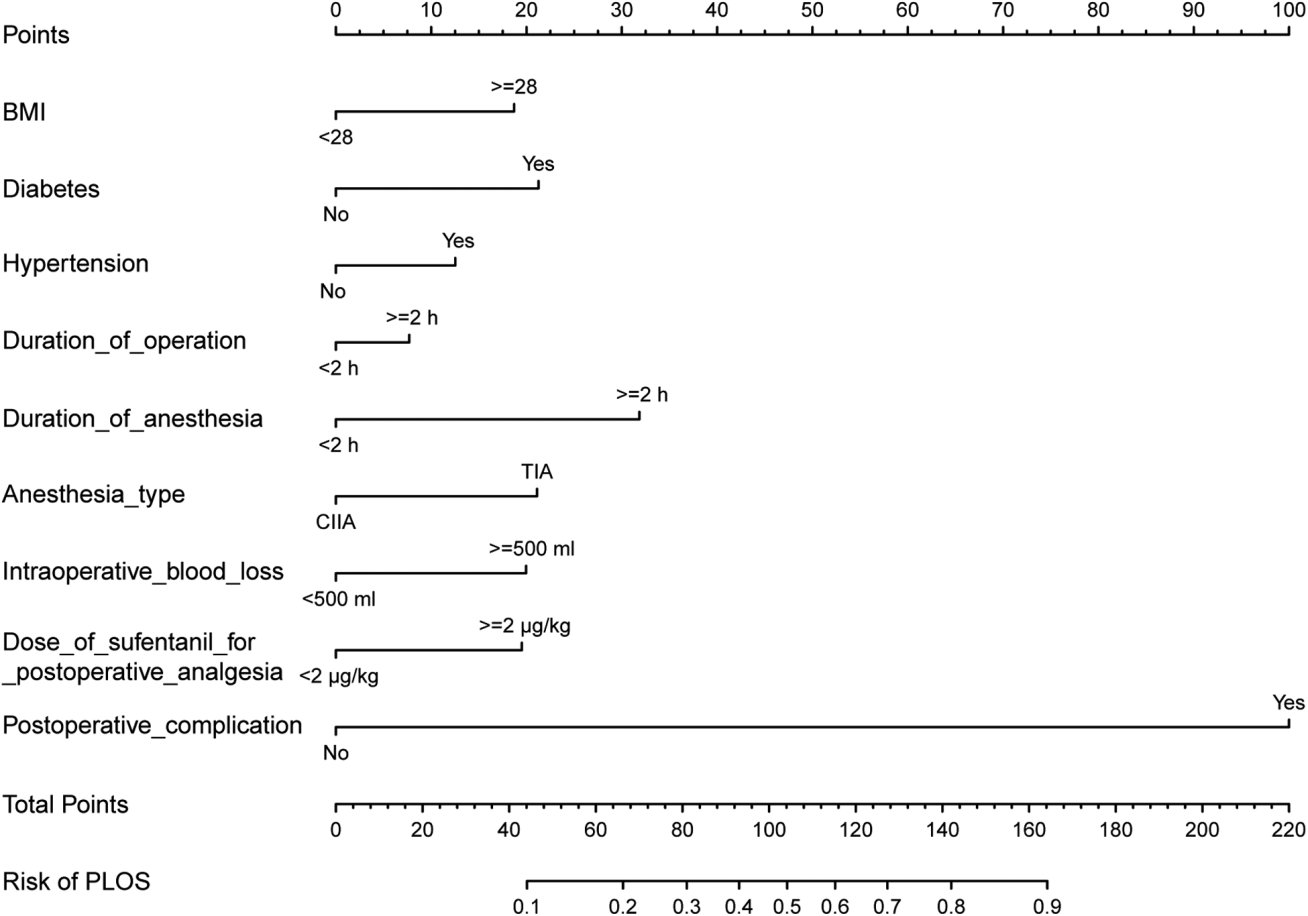
Developing a nomogram for prolonged PLOS risk. The prolonged PLOS risk nomogram was developed in the cohort, with BMI, diabetes, hypertension, duration of operation, duration of anesthesia, anesthesia type, estimated blood loss, dose of sufentanil for postoperative analgesia, and postoperative complication by R software (Version 4.1.3; https://www.R-project.org) with packages (“rms”). PLOS, postoperative length of stay; BMI, body mass index; ASA, American Society of Anesthesiologists; CIIA, combined intravenous and inhaled anesthesia; TIA, total intravenous anesthesia.

The results of the logistic regression analysis among BMI, diabetes, hypertension, duration of surgery, duration of anesthesia, anesthesia type, intraoperative blood loss, sufentanil for postoperative analgesia, and postoperative complication are given in Table 2. The model that incorporated the above predictors was developed and presented as the nomogram (Figure 3).

### Apparent performance of the prolonged PLOS risk nomogram in the cohort

**Figure 4:**
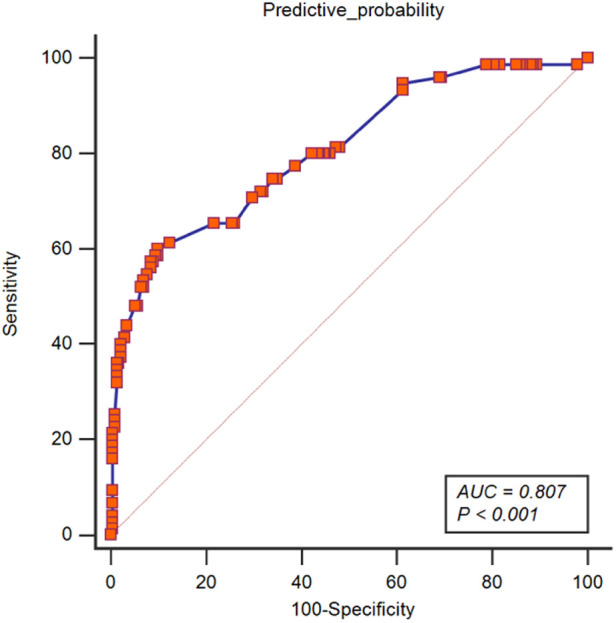
ROC of a predictive nomogram model. The ROC of this model was drawn with the variables of BMI, diabetes, hypertension, duration of operation, duration of anesthesia, anesthesia type, estimated blood loss, dose of sufentanil for postoperative analgesia, and postoperative complication by MedCalc (Version 19.2; https://www.medcalc.org). PLOS, postoperative length of stay; BMI, body mass index.

**Figure 5:**
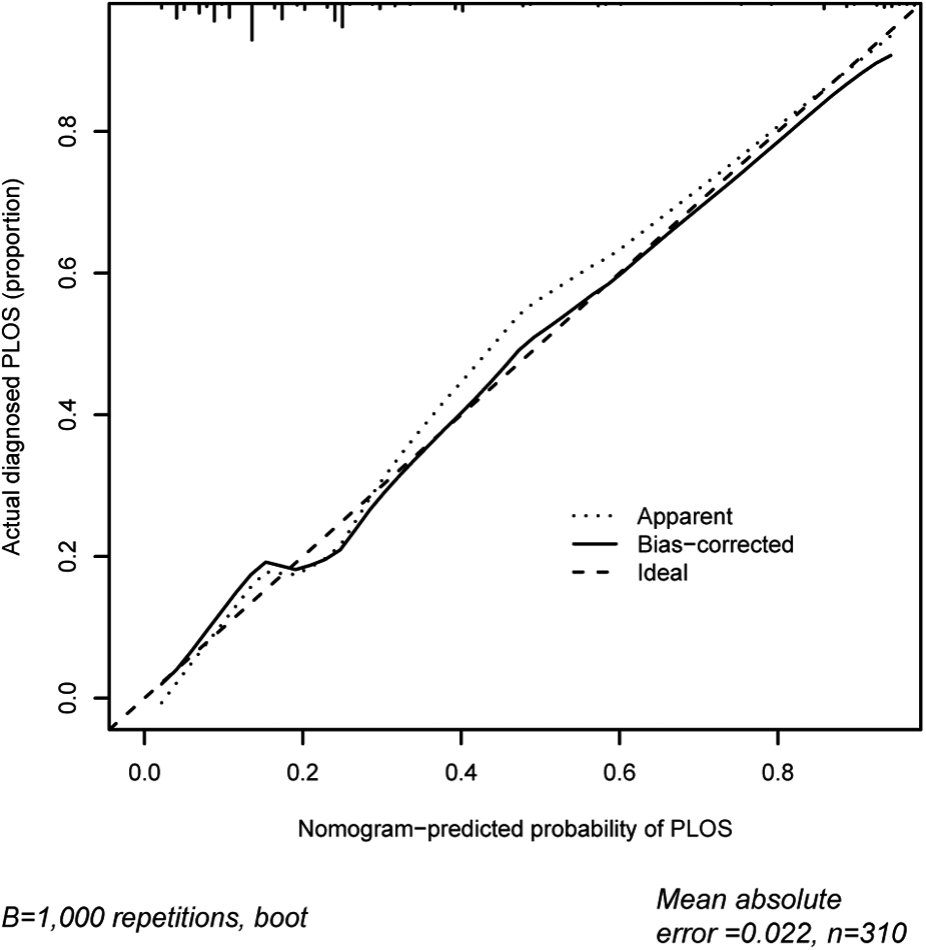
Calibration curves of the prolonged PLOS prediction in the cohort. The *x*-axis represents the predicted prolonged PLOS risk. The *y*-axis represents the actual diagnosed prolonged PLOS. The diagonal dotted line represents a perfect prediction by an ideal model. The solid line represents the performance of the nomogram, of which a closer fit to the diagonal dotted line represents a better prediction.

The area under the ROC curve for the prediction model nomogram was 0.807 (95% CI: 0.758–0.849, *P *< 0.001) for the cohort (Figure 4) and was confirmed to be 0.776 through bootstrapping validation, which suggested the model's good discrimination. The calibration curve of the prolonged PLOS risk nomogram for the prediction of prolonged PLOS risk in lumbar fusion surgery patients demonstrated good agreement in this cohort (Figure 5).

In addition, the prediction model was used to distinguish and classify the prolonged PLOS, and the prediction probability was used as the cutoff value 0.2445. There was no statistical difference between the model predictive ability and the diagnostic criteria in our study (*χ*^2 ^= 0.9245, *P *= 0.3363). The discriminant consistency was tested (Kappa value = 0.0.5186, *P* < 0.001), which ranged from 0.41 to 0.60, indicating that the discriminant ability of the model had good consistency. The Youden index was 53.7%, and the total correct rate was 82.9%, indicating that the model has good predictive efficiency (Table 3).

**Table 3:** Discrimination and classification ability of the prolonged PLOS prediction model in lumbar fusion surgery.

Diagnostic criteria	Prediction		Total	*χ* ^2^	*P*	Kappa value (*P*)	Youden index
	+ (PLOS > 7)	− (PLOS ≤ 7)					
+ (PLOS > 7)	45	30	75				
− (PLOS ≤ 7)	23	212	235				
Total	68	242	310	0.9245	0.3363	0.5186 (*P *< 0.001)	0.5378

PLOS, postoperative length of stay.

### Clinical use of the prediction model nomogram

**Figure 6:**
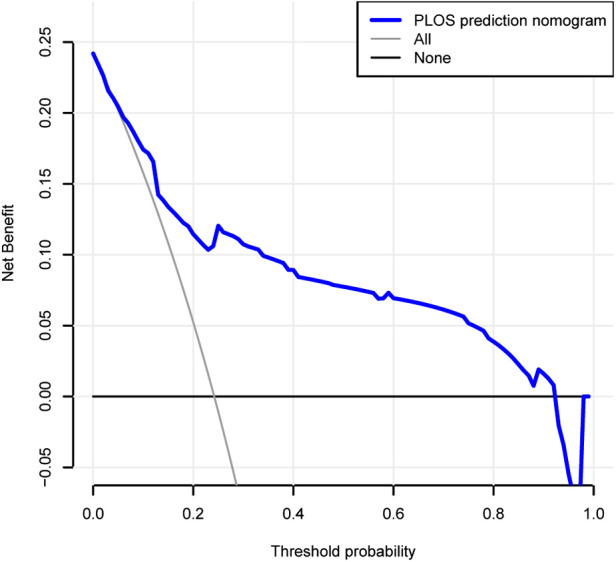
Decision curve analysis for the prolonged PLOS prediction nomogram. The *y*-axis measures the net benefit. The dotted line represents the prolonged PLOS prediction nomogram. The thin solid line represents the assumption that all patients have prolonged PLOS. The thin thick solid line represents the assumption that no patients have prolonged PLOS. The decision curve shows that if the threshold probability of a patient and a doctor is 3% and 92%, respectively, using this prolonged PLOS prediction nomogram in the current study to predict prolonged PLOS risk adds more benefit than the intervention-all-patients scheme or the intervention-none scheme.

The decision curve analysis for the prolonged PLOS nomogram is presented in Figure 6. The decision curve shows that if the threshold probability of a patient and a doctor is >3% and <92%, respectively, using this prolonged PLOS nomogram to predict prolonged PLOS risk adds more benefit than the scheme. Within this range, net benefit was comparable with several overlaps, on the basis of the prolonged PLOS risk nomogram.

## Discussion

In recent years, the concept of enhanced recovery after surgery (ERAS) has been gradually introduced into clinical practice. The length of stay of surgical patients is undoubtedly extremely important to patients and hospitals, but the total length of stay is affected perioperatively by many factors. However, PLOS can more accurately reflect the speed of recovery of patients after surgery, and shortening the PLOS of patients is the core goal of ERAS. Therefore, we developed and validated a novel prediction tool for prolonged PLOS risk among the patients of lumbar fusion surgery by using nine easily available variables. The prediction model nomogram greatly simplifies the complicated operation process and is easy to understand and convenient for clinicians to aid better clinical decision making (21). This was the first study in which a nomogram was applied in lumbar fusion surgery and PLOS. Internal validation in the cohort demonstrated good discrimination and calibration power, suggesting that this nomogram can be widely and accurately used due to its large sample size.

The PLOS of patients is affected by many factors, among which the diagnosis and treatment level of surgeons also plays an important role in addition to the condition of the patients themselves. The PLOS of a certain type of surgery may be different in different medical institutions with different levels of diagnosis and treatment. Therefore, there is no fixed standard for the diagnosis of prolonged PLOS of a certain type of surgery in previous studies. A previous study has shown that if the PLOS data of patients were in accordance with normal distribution, patients with PLOS greater than mean plus 1 standard deviation were defined as prolonged PLOS. For PLOS data that did not conform to normal distribution, patients with PLOS more than P_75_ were defined as PLOS prolongation by calculating median and quartile intervals (P_25_, P_50_, and P_75_) (12, 22). In our study, the PLOS data of 310 patients undergoing lumbar fusion surgery were analyzed, and the results of the normality test showed that they did not conform to the normal distribution. Therefore, we defined patients with PLOS > P_75_ as prolonged PLOS.

We included the variable of postoperative complications in the present study. The results of multivariate regression analysis showed that postoperative complication was an independent risk factor for prolonged PLOS of lumbar fusion surgery. Many postoperative complications are related to the prolonged PLOS of patients, such as postoperative delirium, postoperative cognitive dysfunction, and so on. For anesthesiologists and surgeons, it is necessary to fully predict the possible postoperative complications before surgery and give intervention perioperatively to minimize the occurrence of postoperative complications, so as to facilitate the postoperative recovery of patients and save medical resources. In ERAS, anesthesiologists play an extremely key role, and the management of the peri-anesthetic period is closely related to the postoperative recovery of patients. In the past, anesthesia-related factors were rarely included in PLOS-related studies. Interestingly, in this study, variables such as anesthesia type, duration of anesthesia, and use of sufentanil for postoperative analgesia were included in the analysis of PLOS. Univariate regression analysis found that anesthesia type and duration of anesthesia were associated with the risk of prolonged PLOS, while multivariate regression analysis showed that total intravenous anesthesia (TIA) significantly increased the risk of prolonged PLOS compared with combined intravenous and inhaled anesthesia (CIIA). Therefore, TIA was an independent risk factor for prolonged PLOS in our study and may be used as a predictor of prolonged PLOS. The possible reasons are as follows: there is no monitoring of the depth of anesthesia in this research institution, and there may be excessive depth of anesthesia in the process of TIA, which may affect the postoperative PLOS of patients. In clinical anesthesia, the choice of TIA and CIIA is still controversial. As to whether TIA is related to prolonged PLOS, an analysis of a larger sample size needs to be done, or this aspect should be confirmed by conducting randomized controlled trials.

Previous studies have shown that the duration of surgery is related to the postoperative outcome of patients. Andersen K et al analyzed 335 patients with oral and maxillofacial LeFortI osteotomy. PLOS was defined as the duration from the operation date to the discharge date. The multiple regression model showed that the predictors of PLOS prolongation were duration of surgery and relative blood loss (23). Similarly, another multivariate analysis by Reid Fletcher of 11,430 patients undergoing laparoscopic gastrectomy suggested that prolonged duration of surgery was a predictor of prolonged PLOS (24). However, in our study, the duration of surgery and intraoperative blood loss were not independent predictors for prolonged PLOS. The possible reasons are as follows: (1) the variable definition of intraoperative blood loss is different. In the previous study, the relative blood loss was defined as a variable, while in our study, the amount of blood loss was defined as a binary variable (<500 ml or >500 ml); (2) different types of surgery and different types of blood loss may have different effects on a certain type of surgical PLOS; and (3) the sample size of this study is small and therefore statistically significant results cannot be obtained. In the follow-up study, we will expand the sample size and comprehensively consider the definition standard of variables, which will make our conclusion more reliable.

Many studies have shown that age >65 years old was closely related to prolonged PLOS of spine surgery (10, 22). In our study, age variable was divided into an elderly group (>65 years old) and a non-elderly group (≤65 years old) based on the criteria of WHO diagnosis. However, univariate regression analysis showed that age was not a risk factor for prolonged PLOS. Therefore, our conclusion is inconsistent with the above two studies, and the possible reasons are as follows: (1) The standard for defining prolonged PLOS is different. Jordan et al defined PLOS greater than mean plus a standard deviation as prolonged PLOS, while we defined PLOS greater than 75 percentile (non-normal distribution) as prolonged PLOS. (2) There were different data types when the age variable was included in the regression analysis. Previous studies included age as a continuous variable in the regression analysis, while in our study, the age included in the regression analysis was a binary variable (10, 22). (3) The sample size of our study is still not enough to draw the conclusion that age is a risk factor for predicting prolonged PLOS of lumbar fusion surgery. A further study of expanded sample size and multifaceted analysis will be needed to draw a more reliable conclusion.

Previous studies showed that ASA class ≥3 was significantly associated with prolonged PLOS in patients undergoing hip fracture and colorectal resection surgery (25, 26). However, in our study, ASA class was not associated with prolonged PLOS either in univariate analysis or in multivariate logistic regression analysis. The possible reasons are as follows: (1) the number of patients with ASA 3–4 of this study is relatively small, with only 11 cases (4.7%) and 4 cases (5.3%) of patients in the control group and case group, respectively, which may not have enough impact on the results; and (2) patients with ASA 3–4 may undergo better perioperative treatment. In addition, we found that none of the preoperative comorbidities were associated with prolonged PLOS of lumbar fusion surgery. Then, we identified high-risk patients by combining preoperative comorbidities (three or more), suggesting that the number of preoperative comorbidities was not related to the prolonged PLOS of lumbar fusion surgery. However, in previous studies, the increase in preoperative comorbidities score was related to the prolongation of PLOS (27, 28). Our study failed to conclude that the number of comorbidities was a risk factor for the prolonged PLOS. The possible explanations are as follows: (1) There are many kinds of comorbidities before surgery, and some patients do not undergo a perfect examination, which may omit the diagnosis of some comorbidities and cause bias; (2) In previous studies, the number of three or more comorbidities was usually taken as the critical point, and our study also followed this classification method, which may not reach a statistically significant level because the sample size is small; (3) Comorbidity is a perioperative factor that clinicians, especially anesthesiologists, pay special attention to, which is closely related to the perioperative safety of patients. For patients with preoperative comorbidities, anesthesiologists and surgeons may do a more detailed preoperative follow-up, so as to make a more perfect anesthetic plan.

In the present study, although univariate and multivariate logistic regression analyses showed that many variables were not associated with the increased risk of prolonged PLOS in lumbar fusion surgery, in order to avoid omitting some important clinical variables, we used LASSO regression to screen variables and constructed a prolonged PLOS risk prediction nomogram of lumbar fusion surgery. Additionally, the predictive model had certain predictive discriminant ability and clinical benefits. Prolonged PLOS increased the financial burden of patients and was associated with many perioperative adverse outcomes such as increased hospital-acquired infection and the risk of deep venous thrombosis, even endangering the lives of patients (4, 5). This demonstrates that developing prolonged PLOS risk prediction tools might improve patient outcomes with individualized risk prediction and interventions. We developed a valid prolonged PLOS risk prediction tool, which assisted clinicians with an early identification of patients at a high risk of prolonged PLOS in lumbar fusion surgery.

### Limitations

There are also several limitations in our study. First, our collected data might be only a part representation of lumbar fusion surgery patients. The cohort was not representative of all patients undergoing lumbar fusion surgery. Second, although the robustness of our nomogram was examined extensively with internal validation using bootstrap testing, external validation could not be conducted, and the generalizability was uncertain for other lumbar fusion surgery populations in other regions and countries. It needs to be externally evaluated in wider populations of lumbar fusion surgery. Third, we did not compare it with other machine learning approaches as well, such as support vector, bier score, etc., to understand whether this predictive model had similar AUC in those approaches.

## Conclusion

This study developed a novel nomogram with a relatively good accuracy to help clinicians access the risk of prolonged PLOS in lumbar fusion surgery patients. By an estimate of individual risk, surgeons and anesthesiologists may shorten PLOS and accelerate postoperative recovery of lumbar fusion surgery through more accurate individualized treatment.

## Data Availability

The raw data supporting the conclusions of this article will be made available by the authors, without undue reservation.
